# Collodion in Plastic Operations

**Published:** 1895-08

**Authors:** 


### COLLODION IN PLASTIC OPERATIONS.

In the *Boston Medical and Surgical Journal* for May 23d, 1895 Dr. David Coggin, of Salem, in the correspondence columns, calls attention to the value of collodion in plastic surgery. By its use the inconvenience and pain of sutures can frequently be dispensed with. For example, when inversion of the lower eyelid is present, as is not uncommon in elderly people, and which naturally causes much annoyance, it is often sufficient (Noyes) to apply two or three coats of collodion on the skin below the inturned lashes, and repeat its application every two or three days for some weeks, till either relief follows or the patient consents to have a strip of skin excised. When the latter is done, fibres of cotton saturated with the collodion will keep the edges of the wound in perfect apposition during the healing process, sutures not being called for.

Its contractile property is also of value after the outscooping of cysts on the eyelids. They are ordinarily incised through the tarsal surface, and when evacuated, the sac sometimes becomes much distended by hemorrhage, and occasionally an abscess is the sequel. Collodion applied on the skin over the site of the chalazion expels the blood and brings the walls together, and so promotes immediate union. This is favored by inserting a drop of castor oil in the line of the incision, which it tends to keep open for a day or two. He also again calls attention to the use of collodion on distended tear-sacs, in several instances of which it has helped in abridging the treatment of this not uncommon affection.—*Therapeutic Gazette.*

